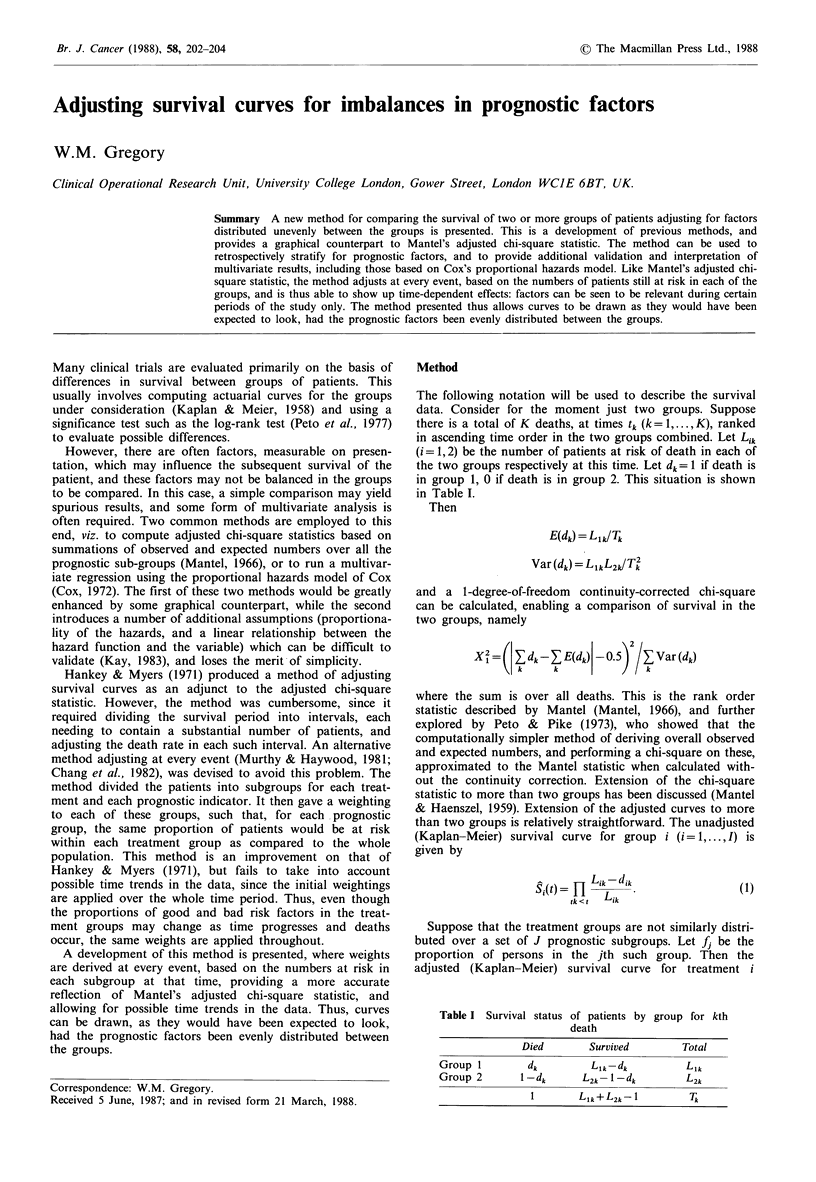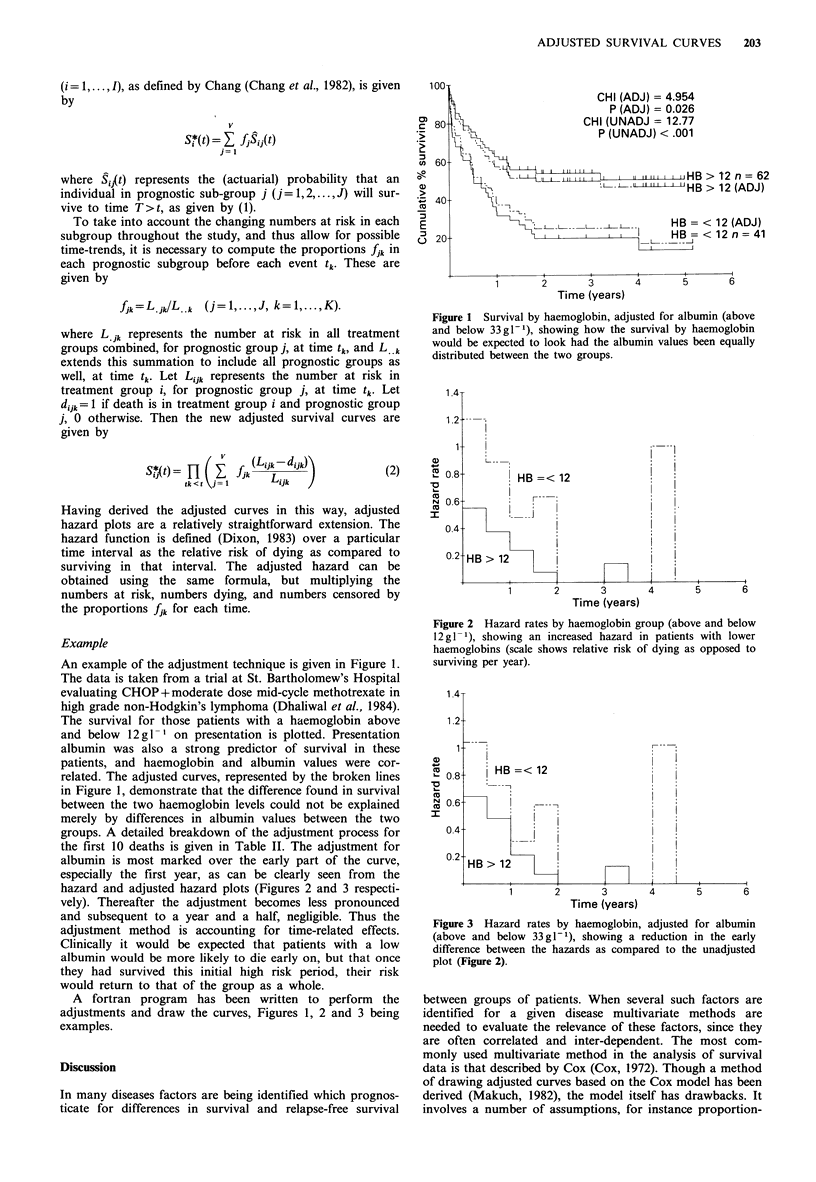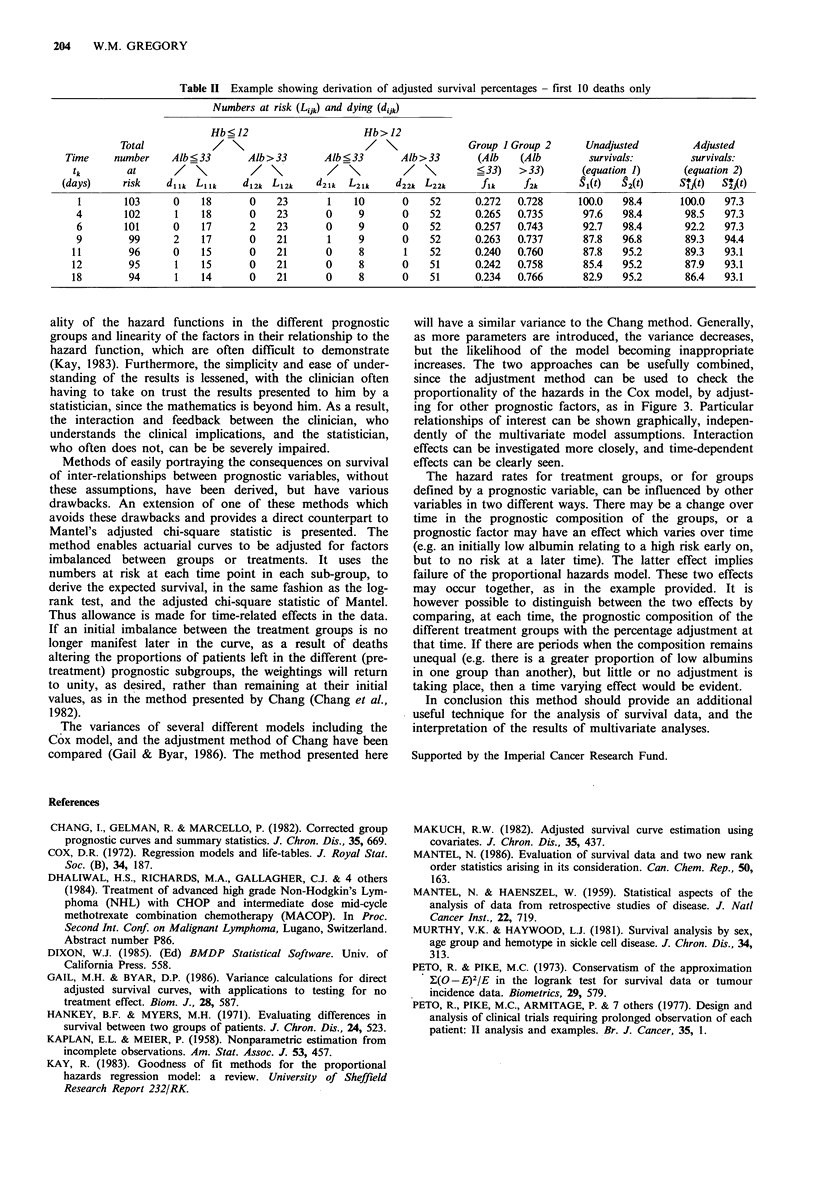# Adjusting survival curves for imbalances in prognostic factors.

**DOI:** 10.1038/bjc.1988.193

**Published:** 1988-08

**Authors:** W. M. Gregory

**Affiliations:** Clinical Operational Research Unit, University College London, UK.

## Abstract

A new method for comparing the survival of two or more groups of patients adjusting for factors distributed unevenly between the groups is presented. This is a development of previous methods, and provides a graphical counterpart to Mantel's adjusted chi-square statistic. The method can be used to retrospectively stratify for prognostic factors, and to provide additional validation and interpretation of multivariate results, including those based on Cox's proportional hazards model. Like Mantel's adjusted chi-square statistic, the method adjusts at every event, based on the numbers of patients still at risk in each of the groups, and is thus able to show up time-dependent effects: factors can be seen to be relevant during certain periods of the study only. The method presented thus allows curves to be drawn as they would have been expected to look, had the prognostic factors been evenly distributed between the groups.


*Br. J. Cancer* (1988), **58**, 202–204 © The Macmillan Press Ltd., 1988

### Adjusting survival curves for imbalances in prognostic factors

W.M. Gregory

*Clinical Operational Research Unit, University College London, Gower Street, London WC1E 6BT, UK.*

**Summary** A new method for comparing the survival of two or more groups of patients adjusting for factors distributed unevenly between the groups is presented. This is a development of previous methods, and provides a graphical counterpart to Mantel's adjusted chi-square statistic. The method can be used to retrospectively stratify for prognostic factors, and to provide additional validation and interpretation of multivariate results, including those based on Cox's proportional hazards model. Like Mantel's adjusted chi-square statistic, the method adjusts at every event, based on the numbers of patients still at risk in each of the groups, and is thus able to show up time-dependent effects: factors can be seen to be relevant during certain periods of the study only. The method presented thus allows curves to be drawn as they would have been expected to look, had the prognostic factors been evenly distributed between the groups.

Many clinical trials are evaluated primarily on the basis of differences in survival between groups of patients. This usually involves computing actuarial curves for the groups under consideration (Kaplan & Meier, 1958) and using a significance test such as the log-rank test (Peto *et al.*, 1977) to evaluate possible differences.

However, there are often factors, measurable on presentation, which may influence the subsequent survival of the patient, and these factors may not be balanced in the groups to be compared. In this case, a simple comparison may yield spurious results, and some form of multivariate analysis is often required. Two common methods are employed to this end, *viz.* to compute adjusted chi-square statistics based on summations of observed and expected numbers over all the prognostic sub-groups (Mantel, 1966), or to run a multivariate regression using the proportional hazards model of Cox (Cox, 1972). The first of these two methods would be greatly enhanced by some graphical counterpart, while the second introduces a number of additional assumptions (proportionality of the hazards, and a linear relationship between the hazard function and the variable) which can be difficult to validate (Kay, 1983), and loses the merit of simplicity.

Hankey & Myers (1971) produced a method of adjusting survival curves as an adjunct to the adjusted chi-square statistic. However, the method was cumbersome, since it required dividing the survival period into intervals, each needing to contain a substantial number of patients, and adjusting the death rate in each such interval. An alternative method adjusting at every event (Murthy & Haywood, 1981; Chang *et al.*, 1982), was devised to avoid this problem. The method divided the patients into subgroups for each treatment and each prognostic indicator. It then gave a weighting to each of these groups, such that, for each prognostic group, the same proportion of patients would be at risk within each treatment group as compared to the whole population. This method is an improvement on that of Hankey & Myers (1971), but fails to take into account possible time trends in the data, since the initial weightings are applied over the whole time period. Thus, even though the proportions of good and bad risk factors in the treatment groups may change as time progresses and deaths occur, the same weights are applied throughout.

A development of this method is presented, where weights are derived at every event, based on the numbers at risk in each subgroup at that time, providing a more accurate reflection of Mantel's adjusted chi-square statistic, and allowing for possible time trends in the data. Thus, curves can be drawn, as they would have been expected to look, had the prognostic factors been evenly distributed between the groups.

Correspondence: W.M. Gregory.
Received 5 June, 1987; and in revised form 21 March, 1988.

### Method

The following notation will be used to describe the survival data. Consider for the moment just two groups. Suppose there is a total of $K$ deaths, at times $t_k$ ($k=1,\dots,K$), ranked in ascending time order in the two groups combined. Let $L_{ik}$ ($i=1,2$) be the number of patients at risk of death in each of the two groups respectively at this time. Let $d_k=1$ if death is in group 1, 0 if death is in group 2. This situation is shown in Table I.

Then

$$E(d_k)=L_{1k}/T_k$$

$$\mathrm{Var}(d_k)=L_{1k}L_{2k}/T_k^2$$

and a 1-degree-of-freedom continuity-corrected chi-square can be calculated, enabling a comparison of survival in the two groups, namely

$$X_1^2=\left(\left|\sum_k d_k-\sum_k E(d_k)\right|-0.5\right)^2\Big/\sum_k \mathrm{Var}(d_k)$$

where the sum is over all deaths. This is the rank order statistic described by Mantel (Mantel, 1966), and further explored by Peto & Pike (1973), who showed that the computationally simpler method of deriving overall observed and expected numbers, and performing a chi-square on these, approximated to the Mantel statistic when calculated without the continuity correction. Extension of the chi-square statistic to more than two groups has been discussed (Mantel & Haenszel, 1959). Extension of the adjusted curves to more than two groups is relatively straightforward. The unadjusted (Kaplan–Meier) survival curve for group $i$ ($i=1,\dots,I$) is given by

$$\hat{S}_i(t)=\prod_{tk<t}\frac{L_{ik}-d_{ik}}{L_{ik}}. \quad (1)$$

Suppose that the treatment groups are not similarly distributed over a set of $J$ prognostic subgroups. Let $f_j$ be the proportion of persons in the $j$th such group. Then the adjusted (Kaplan–Meier) survival curve for treatment $i$

**Table I** Survival status of patients by group for $k$th death

| | *Died* | *Survived* | *Total* |
|---|---|---|---|
| Group 1 | $d_k$ | $L_{1k}-d_k$ | $L_{1k}$ |
| Group 2 | $1-d_k$ | $L_{2k}-1-d_k$ | $L_{2k}$ |
| | 1 | $L_{1k}+L_{2k}-1$ | $T_k$ |

($i=1,\ldots,I$), as defined by Chang (Chang *et al.*, 1982), is given by

$$S_i^*(t)=\sum_{j=1}^{V} f_j\hat{S}_{ij}(t)$$

where $\hat{S}_{ij}(t)$ represents the (actuarial) probability that an individual in prognostic sub-group $j$ ($j=1,2,\ldots,J$) will survive to time $T>t$, as given by (1).

To take into account the changing numbers at risk in each subgroup throughout the study, and thus allow for possible time-trends, it is necessary to compute the proportions $f_{jk}$ in each prognostic subgroup before each event $t_k$. These are given by

$$f_{jk}=L_{.jk}/L_{..k} \quad (j=1,\ldots,J,\ k=1,\ldots,K).$$

where $L_{.jk}$ represents the number at risk in all treatment groups combined, for prognostic group $j$, at time $t_k$, and $L_{..k}$ extends this summation to include all prognostic groups as well, at time $t_k$. Let $L_{ijk}$ represents the number at risk in treatment group $i$, for prognostic group $j$, at time $t_k$. Let $d_{ijk}=1$ if death is in treatment group $i$ and prognostic group $j$, 0 otherwise. Then the new adjusted survival curves are given by

$$S_{ij}^*(t)=\prod_{tk<t}\left(\sum_{j=1}^{V} f_{jk}\frac{(L_{ijk}-d_{ijk})}{L_{ijk}}\right) \qquad (2)$$

Having derived the adjusted curves in this way, adjusted hazard plots are a relatively straightforward extension. The hazard function is defined (Dixon, 1983) over a particular time interval as the relative risk of dying as compared to surviving in that interval. The adjusted hazard can be obtained using the same formula, but multiplying the numbers at risk, numbers dying, and numbers censored by the proportions $f_{jk}$ for each time.

*Example*

An example of the adjustment technique is given in Figure 1. The data is taken from a trial at St. Bartholomew's Hospital evaluating CHOP+moderate dose mid-cycle methotrexate in high grade non-Hodgkin's lymphoma (Dhaliwal *et al.*, 1984). The survival for those patients with a haemoglobin above and below $12\,g\,l^{-1}$ on presentation is plotted. Presentation albumin was also a strong predictor of survival in these patients, and haemoglobin and albumin values were correlated. The adjusted curves, represented by the broken lines in Figure 1, demonstrate that the difference found in survival between the two haemoglobin levels could not be explained merely by differences in albumin values between the two groups. A detailed breakdown of the adjustment process for the first 10 deaths is given in Table II. The adjustment for albumin is most marked over the early part of the curve, especially the first year, as can be clearly seen from the hazard and adjusted hazard plots (Figures 2 and 3 respectively). Thereafter the adjustment becomes less pronounced and subsequent to a year and a half, negligible. Thus the adjustment method is accounting for time-related effects. Clinically it would be expected that patients with a low albumin would be more likely to die early on, but that once they had survived this initial high risk period, their risk would return to that of the group as a whole.

A fortran program has been written to perform the adjustments and draw the curves, Figures 1, 2 and 3 being examples.

### Discussion

In many diseases factors are being identified which prognosticate for differences in survival and relapse-free survival

**Figure 1** Survival by haemoglobin, adjusted for albumin (above and below $33\,g\,l^{-1}$), showing how the survival by haemoglobin would be expected to look had the albumin values been equally distributed between the two groups.

**Figure 2** Hazard rates by haemoglobin group (above and below $12\,g\,l^{-1}$), showing an increased hazard in patients with lower haemoglobins (scale shows relative risk of dying as opposed to surviving per year).

**Figure 3** Hazard rates by haemoglobin, adjusted for albumin (above and below $33\,g\,l^{-1}$), showing a reduction in the early difference between the hazards as compared to the unadjusted plot (**Figure 2**).

between groups of patients. When several such factors are identified for a given disease multivariate methods are needed to evaluate the relevance of these factors, since they are often correlated and inter-dependent. The most commonly used multivariate method in the analysis of survival data is that described by Cox (Cox, 1972). Though a method of drawing adjusted curves based on the Cox model has been derived (Makuch, 1982), the model itself has drawbacks. It involves a number of assumptions, for instance proportion-

**Table II** Example showing derivation of adjusted survival percentages – first 10 deaths only

| Time $t_k$ (days) | Total number at risk | Numbers at risk ($L_{ijk}$) and dying ($d_{ijk}$): Hb≦12, Alb≦33, $d_{11k}$ | Hb≦12, Alb≦33, $L_{11k}$ | Hb≦12, Alb>33, $d_{12k}$ | Hb≦12, Alb>33, $L_{12k}$ | Hb>12, Alb≦33, $d_{21k}$ | Hb>12, Alb≦33, $L_{21k}$ | Hb>12, Alb>33, $d_{22k}$ | Hb>12, Alb>33, $L_{22k}$ | Group 1 (Alb ≦33) $f_{1k}$ | Group 2 (Alb >33) $f_{2k}$ | Unadjusted survivals: (equation 1) $\hat{S}_1(t)$ | Unadjusted survivals: (equation 1) $\hat{S}_2(t)$ | Adjusted survivals: (equation 2) $S^*_{1j}(t)$ | Adjusted survivals: (equation 2) $S^*_{2j}(t)$ |
|---|---|---|---|---|---|---|---|---|---|---|---|---|---|---|---|
| 1 | 103 | 0 | 18 | 0 | 23 | 1 | 10 | 0 | 52 | 0.272 | 0.728 | 100.0 | 98.4 | 100.0 | 97.3 |
| 4 | 102 | 1 | 18 | 0 | 23 | 0 | 9 | 0 | 52 | 0.265 | 0.735 | 97.6 | 98.4 | 98.5 | 97.3 |
| 6 | 101 | 0 | 17 | 2 | 23 | 0 | 9 | 0 | 52 | 0.257 | 0.743 | 92.7 | 98.4 | 92.2 | 97.3 |
| 9 | 99 | 2 | 17 | 0 | 21 | 1 | 9 | 0 | 52 | 0.263 | 0.737 | 87.8 | 96.8 | 89.3 | 94.4 |
| 11 | 96 | 0 | 15 | 0 | 21 | 0 | 8 | 1 | 52 | 0.240 | 0.760 | 87.8 | 95.2 | 89.3 | 93.1 |
| 12 | 95 | 1 | 15 | 0 | 21 | 0 | 8 | 0 | 51 | 0.242 | 0.758 | 85.4 | 95.2 | 87.9 | 93.1 |
| 18 | 94 | 1 | 14 | 0 | 21 | 0 | 8 | 0 | 51 | 0.234 | 0.766 | 82.9 | 95.2 | 86.4 | 93.1 |

ality of the hazard functions in the different prognostic groups and linearity of the factors in their relationship to the hazard function, which are often difficult to demonstrate (Kay, 1983). Furthermore, the simplicity and ease of understanding of the results is lessened, with the clinician often having to take on trust the results presented to him by a statistician, since the mathematics is beyond him. As a result, the interaction and feedback between the clinician, who understands the clinical implications, and the statistician, who often does not, can be be severely impaired.

Methods of easily portraying the consequences on survival of inter-relationships between prognostic variables, without these assumptions, have been derived, but have various drawbacks. An extension of one of these methods which avoids these drawbacks and provides a direct counterpart to Mantel's adjusted chi-square statistic is presented. The method enables actuarial curves to be adjusted for factors imbalanced between groups or treatments. It uses the numbers at risk at each time point in each sub-group, to derive the expected survival, in the same fashion as the log-rank test, and the adjusted chi-square statistic of Mantel. Thus allowance is made for time-related effects in the data. If an initial imbalance between the treatment groups is no longer manifest later in the curve, as a result of deaths altering the proportions of patients left in the different (pre-treatment) prognostic subgroups, the weightings will return to unity, as desired, rather than remaining at their initial values, as in the method presented by Chang (Chang *et al.*, 1982).

The variances of several different models including the Cox model, and the adjustment method of Chang have been compared (Gail & Byar, 1986). The method presented here will have a similar variance to the Chang method. Generally, as more parameters are introduced, the variance decreases, but the likelihood of the model becoming inappropriate increases. The two approaches can be usefully combined, since the adjustment method can be used to check the proportionality of the hazards in the Cox model, by adjusting for other prognostic factors, as in Figure 3. Particular relationships of interest can be shown graphically, independently of the multivariate model assumptions. Interaction effects can be investigated more closely, and time-dependent effects can be clearly seen.

The hazard rates for treatment groups, or for groups defined by a prognostic variable, can be influenced by other variables in two different ways. There may be a change over time in the prognostic composition of the groups, or a prognostic factor may have an effect which varies over time (e.g. an initially low albumin relating to a high risk early on, but to no risk at a later time). The latter effect implies failure of the proportional hazards model. These two effects may occur together, as in the example provided. It is however possible to distinguish between the two effects by comparing, at each time, the prognostic composition of the different treatment groups with the percentage adjustment at that time. If there are periods when the composition remains unequal (e.g. there is a greater proportion of low albumins in one group than another), but little or no adjustment is taking place, then a time varying effect would be evident.

In conclusion this method should provide an additional useful technique for the analysis of survival data, and the interpretation of the results of multivariate analyses.

Supported by the Imperial Cancer Research Fund.

**References**

CHANG, I., GELMAN, R. & MARCELLO, P. (1982). Corrected group prognostic curves and summary statistics. *J. Chron. Dis.*, **35**, 669.

COX, D.R. (1972). Regression models and life-tables. *J. Royal Stat. Soc.* (B), **34**, 187.

DHALIWAL, H.S., RICHARDS, M.A., GALLAGHER, C.J. & 4 others (1984). Treatment of advanced high grade Non-Hodgkin's Lymphoma (NHL) with CHOP and intermediate dose mid-cycle methotrexate combination chemotherapy (MACOP). In *Proc. Second Int. Conf. on Malignant Lymphoma,* Lugano, Switzerland. Abstract number P86.

DIXON, W.J. (1985). (Ed) *BMDP Statistical Software*. Univ. of California Press. 558.

GAIL, M.H. & BYAR, D.P. (1986). Variance calculations for direct adjusted survival curves, with applications to testing for no treatment effect. *Biom. J.*, **28**, 587.

HANKEY, B.F. & MYERS, M.H. (1971). Evaluating differences in survival between two groups of patients. *J. Chron. Dis.*, **24**, 523.

KAPLAN, E.L. & MEIER, P. (1958). Nonparametric estimation from incomplete observations. *Am. Stat. Assoc. J.* **53**, 457.

KAY, R. (1983). Goodness of fit methods for the proportional hazards regression model: a review. *University of Sheffield Research Report 232/RK.*

MAKUCH, R.W. (1982). Adjusted survival curve estimation using covariates. *J. Chron. Dis.*, **35**, 437.

MANTEL, N. (1986). Evaluation of survival data and two new rank order statistics arising in its consideration. *Can. Chem. Rep.*, **50**, 163.

MANTEL, N. & HAENSZEL, W. (1959). Statistical aspects of the analysis of data from retrospective studies of disease. *J. Natl Cancer Inst.*, **22**, 719.

MURTHY, V.K. & HAYWOOD, L.J. (1981). Survival analysis by sex, age group and hemotype in sickle cell disease. *J. Chron. Dis.*, **34**, 313.

PETO, R. & PIKE, M.C. (1973). Conservatism of the approximation $\Sigma(O-E)^2/E$ in the logrank test for survival data or tumour incidence data. *Biometrics*, **29**, 579.

PETO, R., PIKE, M.C., ARMITAGE, P. & 7 others (1977). Design and analysis of clinical trials requiring prolonged observation of each patient: II analysis and examples. *Br. J. Cancer*, **35**, 1.